# Fabrication and Characterization of β-Cyclodextrin/*Mosla Chinensis* Essential Oil Inclusion Complexes: Experimental Design and Molecular Modeling

**DOI:** 10.3390/molecules28010037

**Published:** 2022-12-21

**Authors:** Hong-Ning Liu, Xiao-Xia Jiang, Abid Naeem, Fu-Cai Chen, Lu Wang, Yan-Xia Liu, Zhe Li, Liang-Shan Ming

**Affiliations:** Institute for Advanced Study, Key Laboratory of Modern Preparation of TCM, Ministry of Education, Jiangxi University of Chinese Medicine, Nanchang 330004, China

**Keywords:** β-cyclodextrin, *Mosla Chinensis* ‘Jiangxiangru’ essential oil, experimental design, molecular docking, molecular dynamics, carvacrol

## Abstract

Essential oils (EOs) are primarily isolated from medicinal plants and possess various biological properties. However, their low water solubility and volatility substantially limit their application potential. Therefore, the aim of the current study was to improve the solubility and stability of the *Mosla Chinensis* (*M. Chinensis*) EO by forming an inclusion complex (IC) with β-cyclodextrin (β-CD). Furthermore, the IC formation process was investigated using experimental techniques and molecular modeling. The major components of *M. Chinensis* ‘Jiangxiangru’ EOs were carvacrol, thymol, o-cymene, and terpinene, and its IC with β-CD were prepared using the ultrasonication method. Multivariable optimization was studied using a Plackett-Burman design (step 1, identifying key parameters) followed by a central composite design for optimization of the parameters (step 2, optimizing the key parameters). SEM, FT-IR, TGA, and dissolution experiments were performed to analyze the physicochemical properties of the ICs. In addition, the interaction between EO and β-CD was further investigated using phase solubility, molecular docking, and molecular simulation studies. The results showed that the optimal encapsulation efficiency and loading capacity of EO in the ICs were 86.17% and 8.92%, respectively. Results of physicochemical properties were different after being encapsulated, indicating that the ICs had been successfully fabricated. Additionally, molecular docking and dynamics simulation showed that β-CD could encapsulate the EO component (carvacrol) via noncovalent interactions. In conclusion, a comprehensive methodology was developed for determining key parameters under multivariate conditions by utilizing two-step optimization experiments to obtain ICs of EO with β-CD. Furthermore, molecular modeling was used to study the mechanisms involved in molecular inclusion complexation.

## 1. Introduction

**Figure 1:**
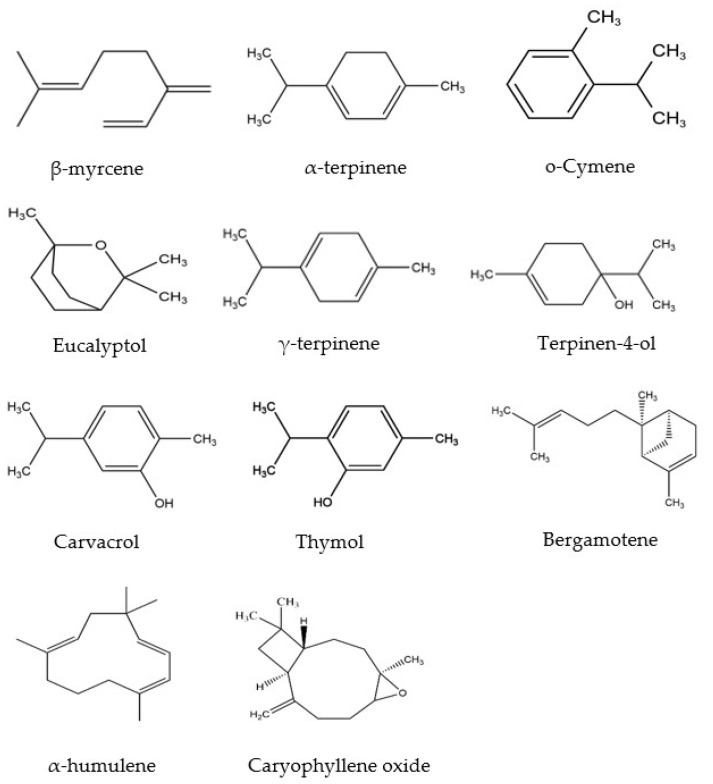
Chemical structures of the main components of *M. Chinensis* EO.

Essential oils (EOs) are naturally occurring volatile components isolated from medicinal plants, exhibiting hydrophobic properties and distinct aromas. EOs have been widely used in the pharmaceutical, cosmeceutical, and food industries for their various biological activities, including antioxidant, anti-inflammatory, antibacterial, sedative, antiparasitic, spasmolytic analgesic activities, etc. [1,2,3,4]. It has been shown that EOs contain different kinds of bioactive compounds, particularly phenolic-derived aromatics and terpenes [5]. Furthermore, recent studies revealed that *Mosla Chinensis* (*M. Chinensis*) ‘Jiangxiangru’ has antioxidant and antibacterial properties, which are related to the phenolic monoterpenes, especially carvacrol [6]. *M. Chinensis* ‘Jiangxiangru’ is widely grown around the world, such as in Vietnam, India, Japan, and especially in the south of China. Since ancient times, this plant has been used for the treatment of diaphoresis and influenza [7]. Our previous research shows that *M. Chinensis* ‘Jiangxiangru’ EO contains various compounds (Figure 1) [7]. However, there are some challenges in using EOs due to their strong volatility, poor thermal stability, and low water solubility. At the same time, the components of Eos are easily oxidized and deteriorated by the environment under oxygen, light, and heat [8,9,10]. Therefore, encapsulating EOs in cyclodextrins (CDs) provides a feasible strategy to enhance the stability, potential, and functional characteristics of EOs [11].

β-cyclodextrin (β-CD) is composed of a seven-membered cyclic oligosaccharide, which has been extensively used in drug delivery systems because of its suitable cavity size of approximately 0.78 nm [11,12]. β-CD consists of a hollow cylindrical ring with a hydrophilic surface mask and hydrophobic cavity, capable of encapsulating EOs by forming inclusion complexes (ICs), resulting in increased water solubility, thermal stability, as well as reducing EO irritation [13,14,15,16]. ICs are formed by various non-covalent forces, including hydrogen bonding, electrostatic, and Van der Waal forces. Ultrasonication technology is commonly used for the preparation of ICs and has several advantages over other mechanical techniques in terms of handling, maintenance, and production costs [17]. The main advantage of ultrasonic technology is its ability to increase mass transfer and phase contact areas due to cavitation and physical phenomena [18]. However, various factors affect the ultrasonic preparation process, such as cyclodextrin amount, EO concentration, and process factors, including power, time, and temperature [19]. Therefore, it is necessary to optimize these parameters through experimental designs in order to achieve effective inclusion complexation.

As a traditional multi-variable optimization, a single-factor experiment presents difficulty in finding the optimal level and is time-consuming. Additionally, the possible interactions among variables are not considered. Recent developments in the design of experiments (DoE), such as response surface methodology, have proven effective not only in saving time and resources but also in improving process efficiency [20,21]. Central composite design (CCD) is an effective method for optimizing and analyzing the interaction between significant parameters through mathematical regression equations [22]. In general, experiments that involve three or more influencing factors are complex and laborious. Therefore, it is necessary to obtain the key variables in a large number of variables prior to analyzing the CCD. The Plackett-Burman design (PBD) is an effective tool to quickly screen the critical variables out from multivariate variables with the least number of observations [23,24]. According to a recent study, β-CD could significantly increase drug release and water solubility in an optimized formulation [25]. Thus, the combination of CCD and PBD allows us to obtain the main factors and provide an in-depth explanation for the preparation of β-CD/EO ICs [26,27].

According to our knowledge and literature searches, there are no reports available on the preparation of ICs of *M. Chinensis* EO with β-CD using both statistical optimization screening and experimental studies involving ultrasonication and molecular modelling to elucidate the process of IC formation. Therefore, in this study, the optimum conditions for the preparation of β-CD/EO IC were studied using factorial (Plackett-Burman design; PBD) and response surface (central composite design; CCD) sequential statistical designs. Moreover, the inclusion mechanism was elucidated through molecular simulation. Specifically, PBD was used to unbiasedly estimate the significant relationship between six variables through a number of experiments. Statistical regression models were constructed and identified using CCD based on the significant variables. Furthermore, the physicochemical properties of EO and β-CD/EO ICs were investigated by scanning electron microscopy (SEM), Fourier transform infrared spectroscopy (FT-IR), thermogravimetric analysis (TGA), and dissolution experiments. In addition, the binding mode between β-CD and EO was determined by a phase solubility study, and the inclusion mechanism was elucidated by molecular docking and molecular dynamics simulation. In summary, a simple and comprehensive method was developed for rapidly screening key factors under multivariate conditions for preparing β-CD/EO ICs. Experimental design coupled with molecular modelling and could be an effective tool for understanding processes and analyzing mechanisms involved in IC formation.

## 2. Results and Discussion

### 2.1. Experimental Designs

#### 2.1.1. Plackett-Burman Design (PBD) Study

The preparation of ICs by ultrasonication technique is influenced by a number of factors. Therefore, it was necessary to screen out the critical quality attributes (such as encapsulation efficiency (Y_1_), loading capacity (Y_2_), etc.) following the principles of pharmaceutical quality by design [28]. PBD is an efficient tool to select and identify the significant factors from multi-factors by comparing the variation analysis among the experimental design results [29].

In this study, 17 runs of PBD with two levels were introduced to unbiasedly screen the significant variables affecting Y_1_ and Y_2_. Table 1 shows the variables analysis of the regression coefficient and *p*-value. Generally, a confidence interval greater than 95% or a significance level less than 0.05 (*p* < 0.05) represents the significant factor [30]. A smaller *p*-value indicates a greater significance of the correlation coefficient; among the 6 variables, A (*p* < 0.005), C (*p* < 0.05), and F (*p* < 0.05) had a significant influence on Y_1_, and A (*p* < 0.01) and F (*p* < 0.05) had a significant effect on Y_2_. As a result, these significant factors (A, C, and F) were investigated for the next optimization stage using CCD [29].

#### 2.1.2. Central Composite Design (CCD) Studies

**Figure 2:**
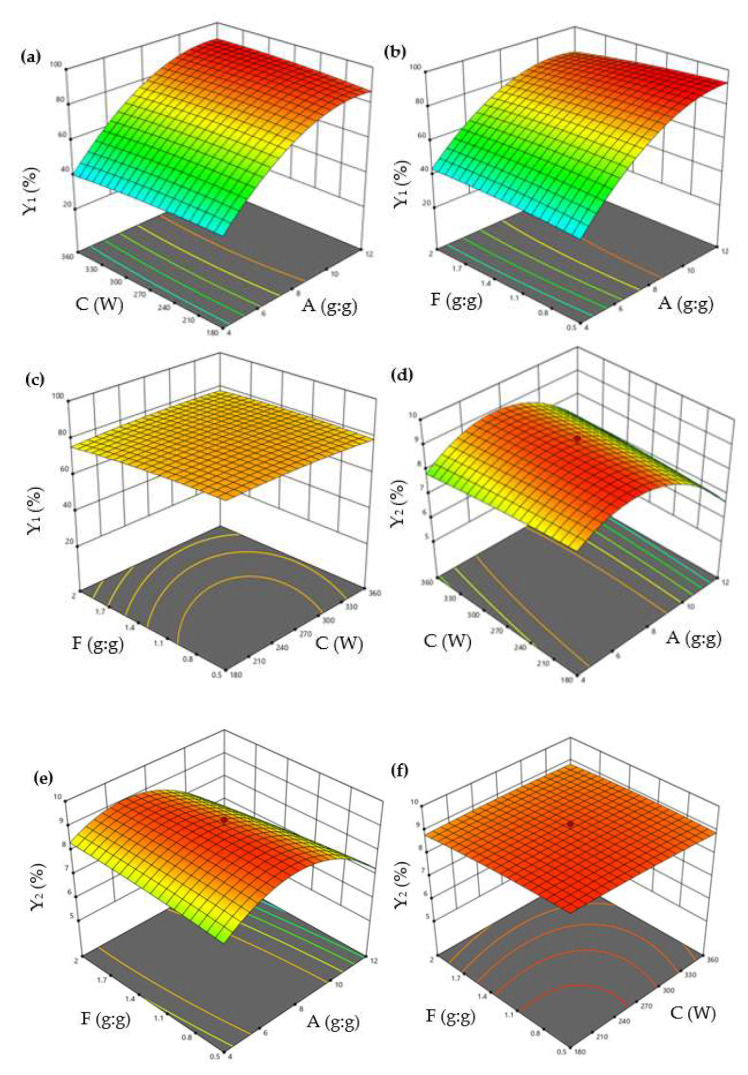
3D surface plots showing interactions between A, C, and F on the Y_1_ and Y_2_. (**a**) The interaction effect of A and C on the Y_1_; (**b**) The interaction effect of A and F on the Y_1_; (**c**) The interaction effect of C and F on the Y_1_; (**d**) The interaction effect of A and C on the Y_2_; (**e**) The interaction effect of A and F on the Y_2_; (**f**) The interaction effect of C and F on the Y_2_. (A: β−CD/EO ratio; C: ultrasonic power; and F: EO/ethanol ratio. Y_1_: encapsulation efficiency and Y_2_: loading capacity).

In the response surface method (RSM), CCD is one of the most commonly used designs, responsible for establishing the model and optimizing the response. The level of CCD optimization factors was designed according to the results of PBD [26,29]. CCD can estimate the interactions between significant factors and optimal level values. The 3D diagrams of the effect of the variables on Y_1_ and Y_2_ are shown in Figure 2. The optimized model for describing Y_1_ and Y_2_ were fitted to a second-order polynomial equation as shown in Equations (1) and (2):

(1) $$Y_{1}=80.758+23.062A-0.109C-1.536F+1.876A*C-2.666A*F+1.216C*F-14.036A^{2}-1.237C^{2}-1.153F^{2}$$

(2) $$Y_{2}=9.073-0.643A-0.062C-0.120F+0.253\;A*C-0.291A*F+0.076C*F-1.466A^{2}-0.054C^{2}-0.078F^{2}$$

Equation (1) revealed that factor A positively impacted Y_1_, while the other two factors (C and F) had a negative impact. Equation (2) revealed that all factors (A, C, and F) had a negative impact on Y_2_.

The positive effect of β-CD concentration (A) on Y_1_ and Y_2_ was attributed to more CD cavities encapsulating the EO with a 1:1 ratio. A study reported that the total polyphenolic content was improved with increasing the β-CD concentration [31]. Moreover, the effect of ultrasonic power (C) on the yield of cholesterol was increased as the power was increased from 0 to 150 W. However, the increasing rate slowed down when the power was increased from 150 to 250 W [32]. Similarly, reports have shown that modest ultrasonic power is beneficial for the formation of ICs [32]. Similarly, in the current study, ultrasonic power had a negative impact and did not show statistically significant changes in Y_1_ and Y_2_. It may be due to the fact that the increase in ultrasonic power caused more violent shock waves and high-speed jets, which easily broke the non-covalent bonds between EO and β-CD [33]. In addition, ethanol increased the inclusion capacity of the CD cavity by forming hydrogen bonds with the hydroxyl groups of the CDs [34]. Nevertheless, when the ethanol concentration reaches a certain amount, the competitive inhibition of EO might occur in the β-CD cavity, which was one of the main reasons that the EO/ethanol ratio (F) negatively affected Y_1_ and Y_2_.

The ANOVA results of the quadratic model Equations (1) and (2) are presented in Table 2. According to the ANOVA results, the regression analysis of the experimental designs indicated that the linear coefficients (A), interaction coefficient (AF), and quadratic coefficients (A^2^) were significant (*p* < 0.05) to Y_1_, and the linear coefficients (A) and quadratic coefficients (A^2^) were significant (*p* < 0.05) to Y_2_. The model fitness was assessed by regression coefficient values (R^2^) and adjusted R^2^ [35]. The value of R^2^ and adj. R^2^ was 0.9906 and 0.9785 in the Y_1_ model, respectively, and the value of R^2^ and adj. R^2^ was 0.9373 and 0.8567 in the Y_2_ model, respectively. These values suggested that both calculated models fitted accurately with the experimental data because of the smaller differences between adj. R^2^ and R^2^, which were less than 0.2 [35].

The interaction of variables and optimum levels (A, C, and F) to Y_1_ and Y_2_ were plotted by constructing the 3D response surface (Figure 2). Figure 2a shows the response surface plots for Y_1_ based on the changes of the β-CD/EO ratio (A) and ultrasonic power (C), while maintaining the EO/ethanol ratio (F) at a constant concentration. The values of Y_1_ increased with A and decreased with C. The interaction effect of the β-CD/EO ratio (A), EO/ethanol ratio (F) on Y_1_, and ultrasonic power (C) kept at the middle concentration were shown in Figure 2b. It could be seen that the value of Y_1_ was the highest at the highest A and the lowest F. The combined effect of ultrasonic power (C) and EO/ethanol ratio (F) on Y_1_ are shown in Figure 2c. The response surface of Y_1_ was elliptical, indicating there might be less interaction between C and F [9]. Similarly, Figure 2d–f showed the interaction effects of the β-CD/EO ratio (A), ultrasonic power (C), and EO/ethanol ratio (F) on Y_2_. The results indicated that both A and C had maximum effects on Y_2_.

#### 2.1.3. Experimental Design Validation

The optimal variables of the encapsulation efficiency (Y_1_) and loading capacity (Y_2_) values were obtained by maximization of the intended values. Maximum Y_1_ and Y_2_ values were predicted at 85.52% and 9.03%, respectively, under the optimal level of β-CD/EO ratio (8.73/g:g), ultrasonic power (213.56/W), and EO/ethanol ratio (0.50/g:g). The optimized experiments were carried out in triplicate. Table 3 shows the predicted values and specific actual experimental results. The mean values of Y_1_ and Y_2_ were 86.17% and 8.92%, and the RD from predicted values were 0.01% and −0.01%, respectively. These results verified that the protocol model had good prediction and reliability.

### 2.2. Physicochemical Characterization

#### 2.2.1. SEM Analysis

**Figure 3:**
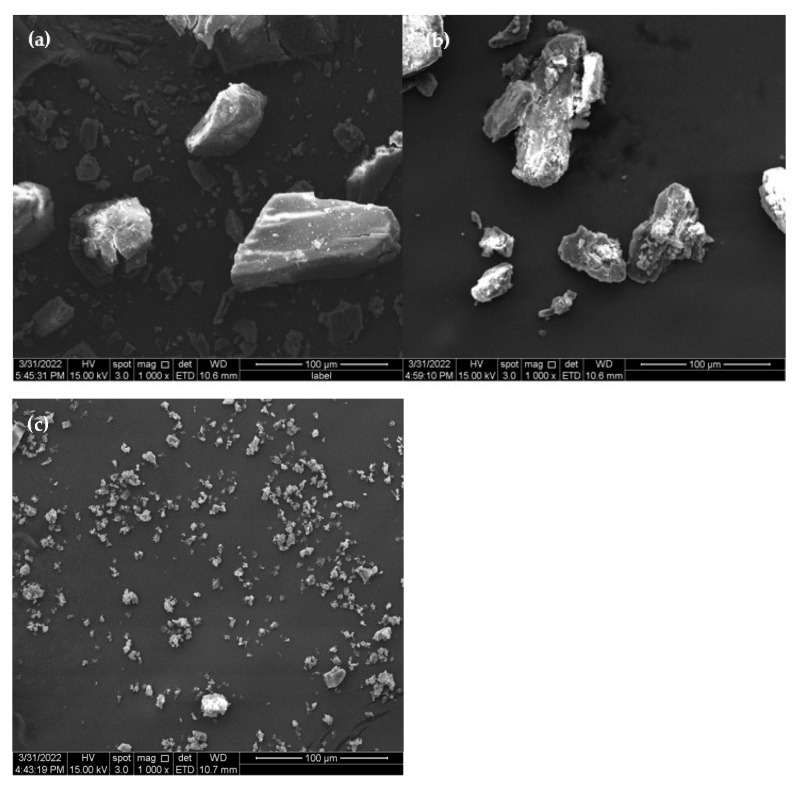
The SEM images. (**a**) β-CD; (**b**) PM; (**c**) β−CD/EO IC.

The surface morphology of the molecule would change in response to interactions between guests (drugs) and CDs (hosts) [9]. The SEM images of β-CD, PM, and β-CD/EO ICs are shown in Figure 3. As shown in Figure 3a, β-CD was composed of smooth blocky particles with irregular shapes. Figure 3b shows the PM image, showing precipitating solids consisting of EO and β-CD with no fixed shape aggregating and adhering to the β-CD surface [36]. The image of β-CD/EO ICs is shown in Figure 3c. The β-CD/EO ICs consisted of irregular shapes and aggregated small particles with smooth surfaces [37]. Compared with β-CD and PM, the size of β-CD/EO ICs showed no visible fractures, cracks, or pores, indicating that the IC was different in morphology formation of a new substance.

#### 2.2.2. FT-IR Analysis

**Figure 4:**
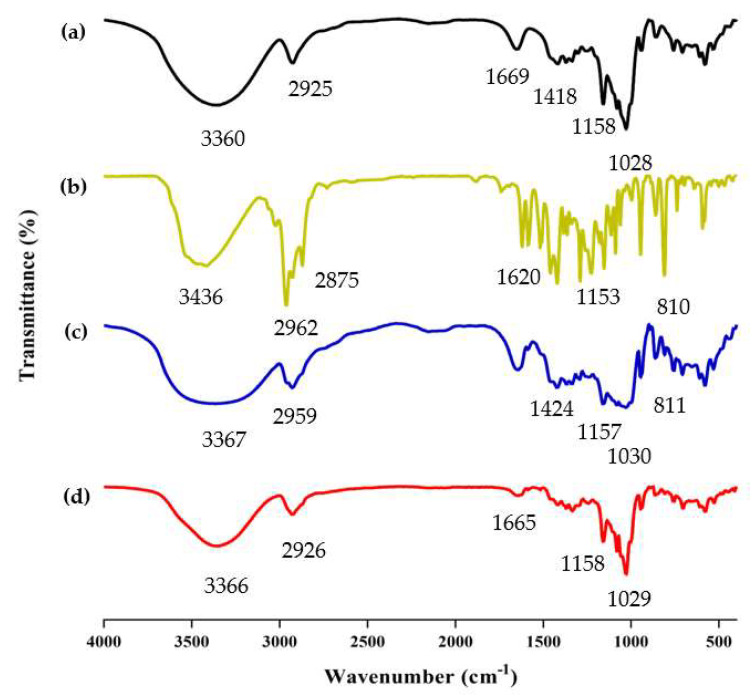
The results of FT−IR spectra. (**a**) β−CD; (**b**) EO; (**c**) PM; (**d**) β−CD/EO IC.

FT-IR analysis is frequently used to study the chemical structure and possible interactions within the ICs [37]. The successful formation of ICs was determined by comparing the differences among β-CD/EO IC, β-CD, EO, and PM, such as the disappearance, displacement, or reduction of absorption peaks [38]. The FT-IR spectrum of β-CD, EO, PM, and β-CD/EO IC within the range of 400 ~ 4000 cm^−1^ wavelength is shown in Figure 4.

The spectrum of β-CD is presented in Figure 4a. The absorption peaks at 3360 cm^−1^ and 2925 cm^−1^ were assigned to O-H and C-H stretching vibrations, respectively [39]. The absorption peak at 1669 cm^−1^ was assigned to H-O-H stretching. The peaks at 1158 and 1028 cm^−1^ represented the vibrations of the C-O-C symmetrical and asymmetrical stretching, respectively [40,41]. Due to the presence of multiple components, the EO shows multiple absorption peaks. As shown in Figure 4b, the EO shows absorption bands in the range of 3200~3500 cm^−1^, which were related to the presence of -OH stretching. The two peaks at 2875 cm^−1^ and 2962 cm^−1^ were the C-H vibrational couplings of -CH_2_. The peaks at 1620, 1584, and 1513 cm^−1^ were assigned to the C=C stretching of the benzene ring of aromatic substances, such as carvacrol and thymol [42]. Compared with β-CD/EO IC, the absorption peaks of PM at 2959 cm^−1^ and 1030 cm^−1^ were redshifted, and the vibration intensity was weakened (Figure 4c). However, the peak intensity of β-CD/EO IC was slightly changed and was similar to β-CD in the range of 400 cm^−1^ to 1500 cm^−1^. Additionally, there were three peaks with weak stretching at 1516~1621 cm^−1^ owing to the bands for EO being obscured by strong and broad β-CD bands (Figure 4d) [43]. Furthermore, the peaks of β-CD/EO IC shifted at 3365 cm^−1^ and weakened at 1165 cm^−1^ [6,44]. Compared with β-CD/EO IC, the characteristic absorption peaks of EO almost totally disappeared at 500~1600 cm^−1^, and there were no new peaks observed. These changes indicated that the β-CD/EO IC had successfully formed via non-covalent bonds, such as hydrogen bonds [45,46].

#### 2.2.3. TGA Analysis

**Figure 5:**
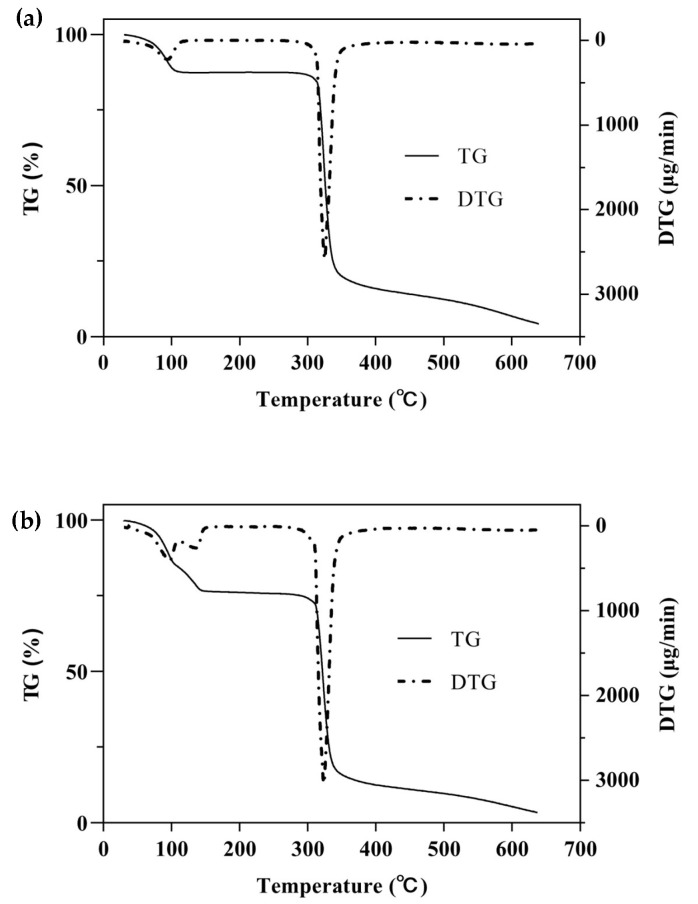

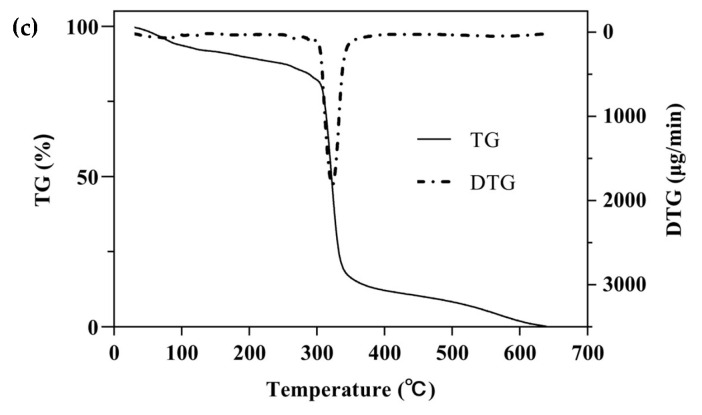
The TGA and DTG curves. (**a**) β-CD; (**b**) PM; (**c**) β-CD/EO IC.

One of the simplest and most commonly used methods for studying the thermal behavior of any formulation or ICs is TG with a continuous increase in temperature [47]. The TGA/DTG curves of β-CD, PM, and β-CD/EO ICs are shown in Figure 5. There were two main mass loss peaks of β-CD, as shown in Figure 5a. The first weight loss was 10.86% from 30 to 100 °C, associated with the evaporation of water molecules in the β-CD cavity [48]. The second weight loss was 67.38% ranging from 270 to 350 °C, and the maximum degradation rate (2.58 mg/min) happened at 325 °C, which was related to the decomposition of β-CD molecules [39,40]. Figure 5b showed three lost peaks of the PM at 96, 135, and 323 °C, which were associated with water evaporation, EO decomposition, and β-CD degradation, respectively [16]. However, the peak of EO volatilization was not observed in the DTG curve of β-CD/EO IC (Figure 5c). At temperatures ranging from 100 to 300 °C, only one decomposition stage of β-CD/EO IC was characterized in the TGA curve with a slight mass loss of 4.42%. In addition, the maximum mass degradation rate was 1.81 mg/min at 322 °C. Furthermore, the DTG curve of β-CD/EO IC showed a peak (1.81 mg/min) at 322 °C lower than β-CD (2.58 mg/min) and PM (3.05 mg/min), indicating that the thermal stability of EO increased and there were no adverse interactions between EO and β-CD [49]. These results suggested that the β-CD/EO IC was effectively fabricated, and the thermal stability was improved, which was consistent with previous studies [50].

#### 2.2.4. In Vitro Dissolution Analysis

**Figure 6:**
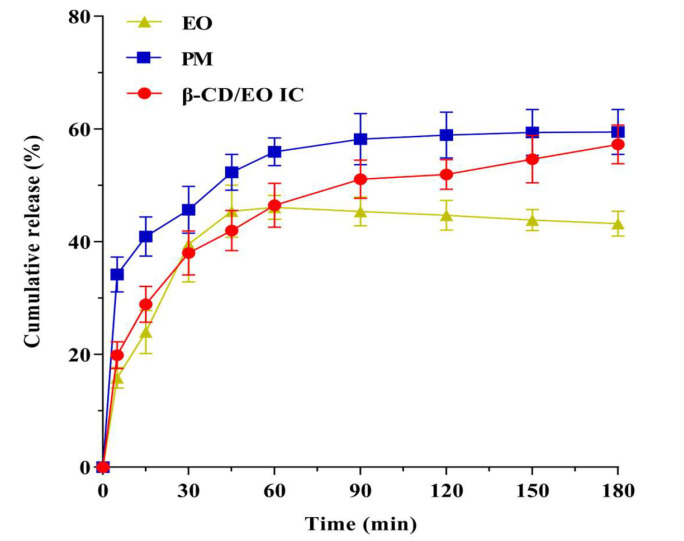
Release curves of β−CD/EO IC, PM and EO in phosphate buffer.

The results of dissolution profiles of EO, PM, and β-CD/EO IC are shown in Figure 6. According to the results, the release rate of EO increased gradually over time. The cumulative release amount of EO was 45.42% within 180 min, and the release reached equilibrium at 45 min. The release behavior of PM was similar to EO, and the maximum release was 55.98% within 60 min. On the contrary, the equilibrium time of β-CD/EO IC was 150 min, and the maximum cumulative release was 54.66%. These results indicated that β-CD/EO IC had slower release characteristics than EO and PM, which could prolong the EO’s curative effect. Therefore, it can be concluded that the bioactivity of the volatile components can be preserved by forming IC with β-CD.

The dissolution mechanism was associated with the binding ability of EO to β-CD, including nonspecific and specific binding [9]. The nonspecific binding was also named physical adsorption. As shown in Figure 6, a certain amount of EO was released quickly from β-CD/EO IC in the first 30 min, which was attributed to the physical contact formed by nonspecific binding, which is more susceptible to being influenced than the strong binding force in the cavity with continuous agitation [49]. In the latter time, from 30 to 180 min, the release of EO was slow and became more stable, which was related to the EO appropriately encapsulated within the cavity of β-CD through the intramolecular hydrogen bonds and Van der Waals forces. In this study, the dissolution behavior of EO was consistent with the previously reported studies, in which flavonoids had different degrees and rates of dissolution after being encapsulated by β-CD [51].

### 2.3. Phase Solubility Study

**Figure 7:**
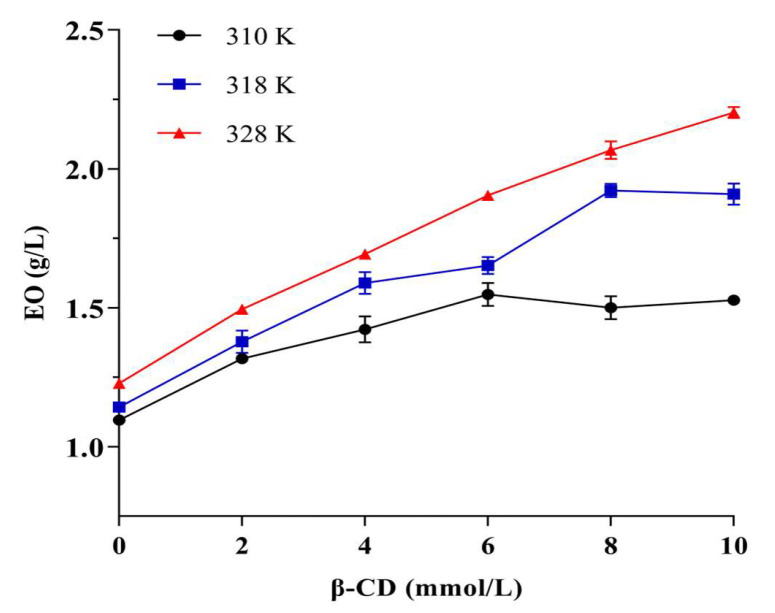
The result of phase solubility study.

Phase solubility studies are the most common methods for determining the ability of macromolecules to interact with small molecules [52,53]. Figure 7 shows the results of the phase solubility study, in which the amount of EO increased with the increase of β-CD concentration and the temperature and reached a status of equilibrium. In this study, there were plateaus at 310 and 318 K at phase solubility curves, while the relationship was linear at 328 K, indicating that the temperature would affect the inclusion ratio of β-CD to EO. The possible reason for this phenomenon was that the *M. Chinensis* EO components were complex, and different components showed a competitive and synergistic relationship with CD, as in our previous study [54]. Generally, a relatively high temperature could enhance the hydrophobic interactions between the molecules [55]. A moderate high temperature was attributed to the increase the complexation of EO in β-CD.

### 2.4. Molecular Docking Analysis

**Figure 8:**
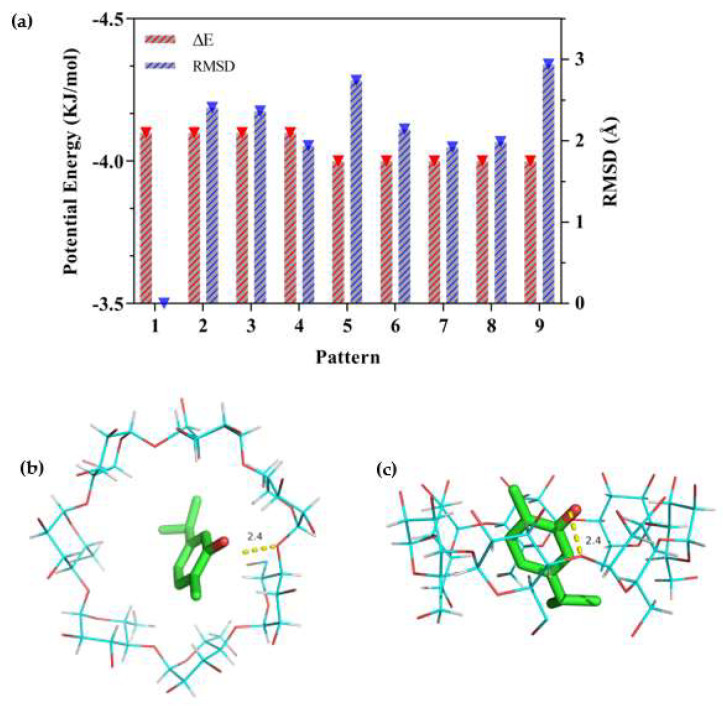
Docking scores of carvacrol in the inner cavity of β−CD. (**a**) The different values of ΔE and RMSD at nine patterns; (**b**) Docked pose of best−ranked docking score (top view); (**c**) Docked pose of best−ranked docking score (side view).

Molecular simulation technology provides an effective method to study the interactions and structures between hosts and guests in a CD system [15,50]. Molecular docking is a powerful way to investigate the inclusion mechanisms at the molecular level and provides the possible inclusion mode [37]. The possible mode is determined by geometric and energy complementarity principles, which ultimately determine the change in binding free energy during the formation of the complex [15]. In the present study, molecular docking was conducted by AutoDock Vina to obtain a visual image of carvacrol with β-CD (Figure 8).

As the docking process was simulated by AutoDock, the ΔE and RMSD values were used to estimate the docking results. The larger the absolute value of ΔE, the more stable the inclusion structure would be, and the smaller the RMSD, the greater the validity of the confirmation [56]. The results of ΔE and RMSD values are shown in Figure 8a, in which the first pattern configuration was selected for the lowest energy and the largest population [57]. Figure 8b and c show the lowest stable energy conformation combined with carvacrol and β-CD. It could be observed that carvacrol was embedded in the β-CD cavity but was not perpendicularly positioned in the symmetry axis of β-CD to allow the maximum amount of H-bonding (2.4 Å) between the molecules. The docking result was consistent with the changes in the FT-IR spectra of the β-CD/EO IC. Pattern 1 in Figure 8a showed that the best-calculated docking score of carvacrol and β-CD was −4.1 kcal/mol, which indicated a strong binding affinity, including hydrophobicity, polarity, repulsion, entropy, and solvent action [51]. A higher negative docking score indicates a more favorable complexation process [58]. Thus, the hydrophobic force was driven by entropy changes, which was in agreement with phase solubility results.

### 2.5. Molecular Dynamics Simulation Analysis

**Figure 9:**
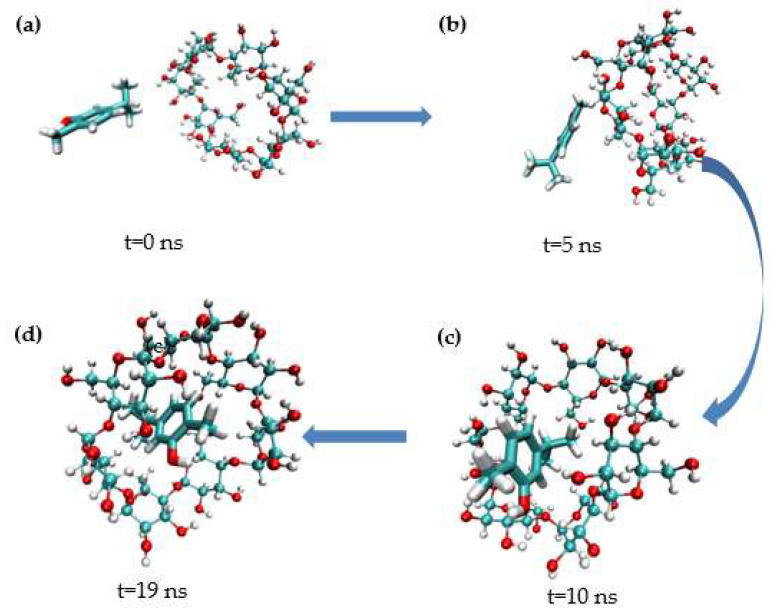
Representative images from carvacrol and β−CD simulation. (**a**) t = 0 ns; (**b**) t = 5 ns; (**c**) t = 10 ns; (**d**) t = 19 ns.

MD simulation studies were carried out to better comprehend the inclusion of carvacrol and β-CD. As shown in Figure 9, the representative conformational changes of the complex structure were intercepted from the simulated initial (0 ns) to stable (19 ns). In the simulation images, carvacrol entered the β-CD cavity via the head side. Carvacrol reached a stable structure through hydrogen bonding at the same location, as shown in the docking analysis. The phenyl ring of carvacrol stacks over the glucopyranose ring of β-CD, demonstrating hydrophobic interactions in which the complex can spontaneously form between carvacrol and β-CD [59].

**Figure 10:**
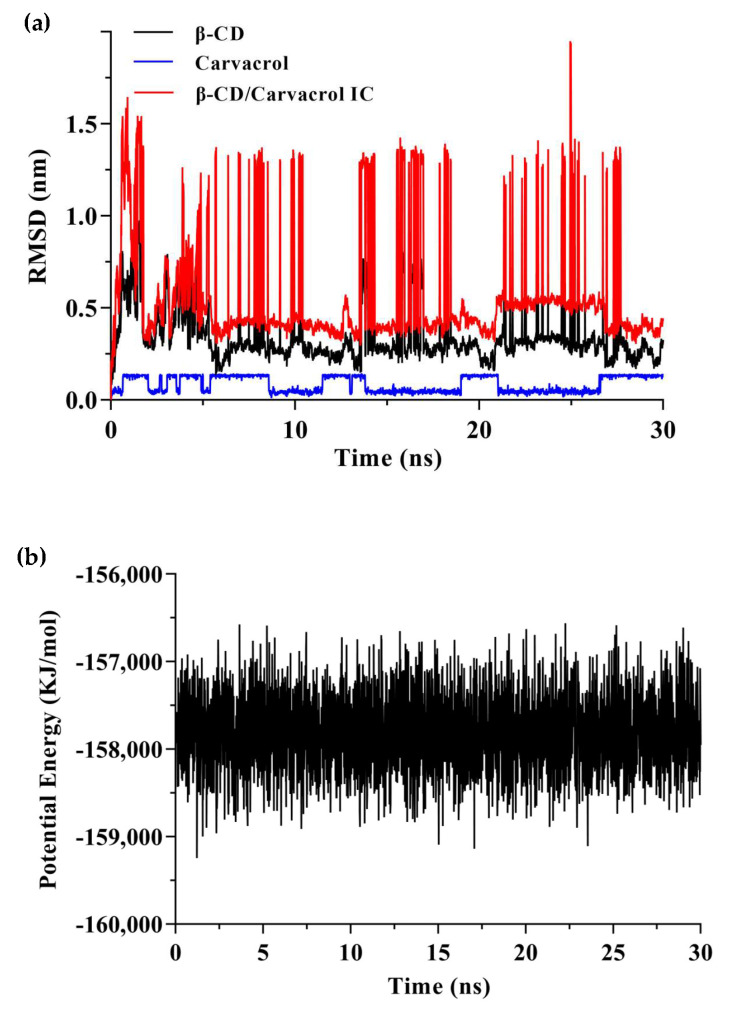
The results of molecular dynamics simulation. (**a**) The RMSD of the β−CD/carvacrol IC during 30 ns simulation; (**b**) The variation in potential energy of the β−CD/carvacrol IC during 30 ns simulation.

Trajectory analysis of the molecular dynamics process was performed to explore the position of carvacrol bounding to β-CD in different timescales (Figure 10). The RMSD for the carvacrol, β-CD, and their complex were plotted individually, as shown in Figure 10a. According to the RMSD plot analysis, there was a dynamic equilibrium between the guest and host molecules. β-CD was a rigid skeleton with a small RMSD value (0.35 nm). The RMSD (0.56 nm) of the complex was larger than β-CD because the carvacrol molecule was encapsulated in the cavity resulting in structural changes. The binding structure of the complex could be considered stable when the RMSD fluctuates within a small range [48]. Furthermore, Figure 10b shows that the potential energy has no abrupt variation, which indicates a supportive dynamic equilibrium in carvacrol and β-CD [60]. Moreover, one carvacrol was bound to one β-CD, resulting in a steric hindrance effect for the volume of the β-CD cavity and the size of the guest molecules.

## 3. Materials and Methods

### 3.1. Materials

β-CD (>98%, Lot#G2007081, Lot#J2114328) and KBr (>99.9%, Lot#H2112256) were provided by Shanghai Aladdin Biochemical Co., Ltd. *M. Chinensis* ‘Jiangxiangru’ was obtained from Jiangzhong Traditional Chinese Medicine Pieces Co., Ltd. Sodium hydroxide was supplied by Sinopharm Reagent. Ethanol (>99.7%) and monopotassium phosphate were purchased from Xilong Scientific Co., Ltd. Deionized water was used throughout the study.

EO was isolated from *M. Chinensis* ‘Jiangxiangru’ herb by hydrodistillation using a Clevenger apparatus, and the EO isolation yield was 1.20%. As our previous study had described, the components of the EO were analyzed by gas chromatography, and mass spectrometry (GC-MS) and results showed that the main components were carvacrol (75.4%), thymol (11%), o-cymene (5.14%), and γ-terpinene (1.31%) [7].

### 3.2. Preparation of ICs

β-CD/EO ICs were prepared under different preparation conditions using the ultrasonication method. Based on previous research [39], β-CD/EO ratio (A/g:g), ultrasonic time (B/min), ultrasonic power (C/W), temperature (D/°C), reaction water (E/mL:g), and EO/ethanol ratio (F/g:g) were selected as the influencing factors. Initially, 30 mL of deionized water was measured with a graduated cylinder, and then a certain amount of β-CD was weighed over a ME303E balance (Mettler—Toledo Instruments Co., Ltd., Shanghai, China). Next, different proportions of EO and ethanol were added slowly to the above solution, followed by ultra—sonication at different temperatures (40, 50, or 60 °C), powers (180, 270, or 360 W), and times (15, 30 or 45 min). Finally, the white β-CD/EO ICs were obtained after allowing the solution to stand at a low temperature for 24 h and then drying it in an oven at 40 °C for 72 h. The β-CD/EO ICs were stored in a desiccator with granular silica gel at 25 °C for subsequent use.

The physical mixture (PM) was prepared at the optimal conditions based on the optimization results of the central composite design, in which the mass of EO and β-CD were weighted out at a specified proportion (8.73:1) and mixed homogeneously for subsequent use.

Encapsulation efficiency (Y_1_) and loading capacity (Y_2_) of EO in ICs were measured using the alcohol extraction method. About 10 mg of β-CD/EO IC was extracted with 10 mL of ethanol for 30 min in an JY92-IIDN ultrasonic bath (Ningbo Xinyi Ultrasonic Equipment Co., Ltd., Ningbo, China), and then filtered by 0.22 μm filter membrane. The content of Y_1_ and Y_2_ was calculated using a UV-spectrophotometer (UV-2600, Japan) at a wavelength of 277 nm, and the concentration of EO was calculated using the established standard curve equation (Y = 0.0147X (μg/mL) − 0.0292, R^2^ = 0.9992). Y_1_ and Y_2_ were calculated using Equation (3) and (4) [61], respectively:

(3) $$Y_{1}(\%)=\frac{m_{1}}{m_{2}}*100$$

(4) $$Y_{2}(\%)=\frac{m_{1}}{m_{3}}*100$$

where m_1_ refers to the mass of encapsulated EO, m_2_ is the mass of total EO added initially, and m_3_ is the total mass of β-CD/EO IC.

### 3.3. Experimental Designs

#### 3.3.1. Step 1: PBD for Screening Key Variables

The PBD experiments were designed using JMP software (version 10.0.0, JMP Statistical Discovery), which generated 15 runs based on 6 variables (A, B, C, D, E, and F). The experiment value was set for each factor at two levels: high level (+1) and low level (−1). Moreover, the center point was set at zero level (0) [20]. Table 4 shows the matrix and corresponding variables of the PBD. Table 5 shows the specific experimental sequence and coding as well as the experimental designs and actual response values. The test results and regression coefficients were analyzed by analysis of variables (ANOVA) to select the significant factors (*p* < 0.05) for the next step of the experimental design.

#### 3.3.2. Step 2: CCD Optimization

The experimental values with significant factors for Y_1_ and Y_2_ were analyzed and optimized by CCD. Y_1_ is the main response value that significantly influences the inclusion outcome [58]. In CCD, significant factors were set at five levels (−α, −1, 0, +1, and +α, where α = 1.287), and non-significant factors were maintained at the zero level [27,62]. The details of the variable labels of CCD are shown in Table 6. The specific codes and the response results of experiments are shown in Table 7 as well as the experimental designs and actual response values. The second-order polynomial equation was fitted on the results of the CCD optimization experiment. The relationship between independent variables and the response value was calculated using Equation (5) [20,27].

(5) $$Y=\beta_{0}+\sum_{i=1}^{3}\beta_{i}X_{i}+\sum_{i=1}^{3}\beta_{ii}X_{ii}{}^{2}+\sum_{i=1}^{3}*\sum_{j=i+1}^{3}\beta_{ij}X_{i}X_{j}$$

where Y, *β_0_*, *β_i_*, *β_ii_*, and *β_ij_* represented the experimental response, intercept term, linear coefficient, square coefficient, interaction and coefficient, respectively. *X_i_* and *X_j_* were independent factors.

#### 3.3.3. Experimental Design Validation

According to the experimental results of CCD, the binomial regression model was used to optimize the response values (encapsulation efficiency (Y_1_) and loading capacity (Y_2_)) based on the optimization module. The maximum willingness mode was utilized to predict the range of optimization parameters. The ANOVA tests between predicted and actual results were conducted to verify whether there were significant differences. Each actual test was conducted in triplicate. Then, the relative deviation (RD) [63] was used to verify the reliability of the optimization parameter combination, and the RD was calculated using Equation (6):

(6) $$\mathrm{Relative}\;\mathrm{Deviation}\;\left(\%\right)=\frac{Predicted\;value-Actual\;value}{Predicted\;value}*100$$

### 3.4. Physicochemical Characterization

To characterize the physical and chemical properties of the β-CD/EO ICs prepared under the optimal preparation conditions the β-CD/EO ratio, ultrasonic power, and EO/ethanol ratio were 8.73:1 (g:g), 213.56 (W), and 1:2 (g:g), respectively. Scanning electron microscopy (SEM), Fourier transform infrared spectroscopy (FT-IR), thermogravimetry analysis (TGA), and in vitro dissolution experiments were conducted on EO, β-CD, β-CD/EO IC and PM.

#### 3.4.1. SEM Determination

The morphology of β-CD, PM and β-CD/EO IC was investigated by FEI Quanta 250 (Oxford Instruments, Czech) scanning electron microscope. Before measurement, each sample was sputtered with gold plating to increase the electrical conductivity and imaged under 15 kV acceleration voltage. The SEM images were collected under a certain magnification [42].

#### 3.4.2. FT-IR Determination

Infrared spectra of the samples were obtained at room temperature using Fourier-transform infrared (FT-IR) spectroscopy (PerkinElmer, UK). β-CD, PM, and β-CD/EO IC with KBr at a ratio of 1:100 were ground and mixed thoroughly together, and two drops of EO were attached to the surface of the KBr tablet, in which the samples were tested at the wavenumber range of 400 ~ 4000 cm^−1^ and the scanning speed was 16 cm^−1^/s. The FT-IR spectral data were recorded after the baseline had been smoothed and corrected by the built-in software [64].

#### 3.4.3. TGA Determination

The thermal properties of β-CD, PM, and β-CD/EO IC were investigated by Exstar TG/DTA 6300 TG analyzer (SII Nano, Japan). In each case, about 5~10 mg of sample was heated from 30 to 700 °C under a 200 mL/min nitrogen atmosphere with a heating rate of 10 °C/min. The thermal behavior of samples was analyzed by thermogravimetry (TG) and derivative thermogravimetry (DTG) curves [64].

#### 3.4.4. In Vitro Dissolution Study

The in vitro dissolution tests were carried out to determine the dissolution behavior of β-CD/EO IC using a ZRS-8G intelligent dissolution apparatus (Tiandatianfa Technology Co. Ltd., Tianjin, China). A certain amount of EO (about 37.30 mg), PM, and β-CD/EO IC (containing an equivalent amount of EO) were separately added to 500 mL phosphate buffer solution (pH = 6.8) at 37 ± 0.5 °C, and then, the rotation speed was set at 50 rpm [65,66]. At predetermined time intervals, 5 mL of the dissolution solution was taken out and centrifuged at 4000 rpm for 5 min. Subsequently, the fresh buffer with the same temperature and volume was replenished to keep the volume constant. The EO concentration in the solution was assayed at 277 nm using a UV spectrophotometer.

### 3.5. Phase Solubility Study

Phase solubility study is widely used to evaluate the interactions between the guest molecule and host molecule at different concentrations [67]. Briefly, the excess EO (300 μL) was added to 5 mL aqueous solution with a series of β-CD solutions ranging from 0 to 10 mmol/L. Moreover, the β-CD/EO suspensions were placed into a heating kettle with a stirring device for 48 h to achieve equilibrium at three temperatures (310, 318, and 328 K). Then, it was filtered through 0.22 μm filters, and the EO absorbance was determined at a wavelength of 277 nm via UV-spectrophotometer. The amount of EO in the solution was plotted against β-CD quantity [68].

### 3.6. Molecular Docking Study

To determine the most possible conformation between EO and β-CD, the interactions were explored using carvacrol, which is the main component found in EO. The complex between carvacrol and β-CD was generated, and the docking fraction was calculated by AutoDock Vina (version 1.5.7) [69]. In the first step, the crystal structure of β-CD was obtained from Cambridge Crystallographic Data Centre (CCDC) and the CCDC ID was 1,235,577 [48]. The 3D structure of carvacrol was downloaded from PubChem (CID 10,364). Initially, the β-CD was set as rigid acceptor molecules, and the carvacrol was used as the ligand molecule that was allowed for flexible twisting [70]. The three-dimensional grid box with 40*40*40 Å size was created and calculated by AutoGrid. Then, 10 simulations with other default parameter settings were performed by Autodock Vina based on the Lamarckian genetic algorithm (LGA) [71].

The docking results were estimated according to binding energy (ΔE) parameters and root mean square deviation (RMSD) values. ΔE represented the energy released, which was mainly used to judge the tightness of the inclusion structure. The RMSD was a deviation statistic that evaluated the stability of the simulation system, which represented the structural changes and atomic fluctuations at the initial position [72].

### 3.7. Molecular Dynamics Simulation Study

Molecular dynamics (MD) simulation was performed to understand the mechanism of the carvacrol encapsulation in the β-CD. MD calculations were performed with the Gromacs molecular dynamic simulation package (version 2021.1) [59]. The visual molecular dynamics (VMD) software was used for all molecular visualizations [73]. The RMSD of all atoms was calculated based on the initial conformations. The Antechamber program was used to generate carvacrol and β-CD force field parameters, and the tLeap (Amber 18.0 Module) was used to generate the molecular topology.

The CHARMM27 force field parameters were used for both molecules, and the system was solvated using the TIP3P model [13]. The system containing a total of 4000 water molecules, 1 molecule of carvacrol and 1 molecule of β-CD, was used for the simulation. The steepest descent method was used to minimize the system’s energy, and the NPT ensemble was performed with 100 ps. The simulation was run for 30 ns using a 2 fs time step for the production. The V-rescale method was used for temperature coupling, and the Parrinello-Rahman method was used for pressure coupling. The particle mesh ewald (PME) method was used for long-range electrostatic interactions. A 14 Å cut-off was employed for Van der Waals force (VdW) and short-range coulombic interactions.

## 4. Conclusions

In summary, two-step experimental designs and molecular dynamics simulation were utilized to investigate the formation and stabilization mechanisms of ICs. Three critical variables, which have an impact on the formation of β-CD/EO IC were obtained from six parameters by PBD and optimized the levels using CCD, including the β-CD/EO ratio, ultrasonic power, and EO/ethanol ratio. The optimum response values of encapsulation efficiency (86.17%) and loading capacities (8.92%) were obtained at 8.73:1 (g:g) of β-CD/EO ratio, 213.56 (W) of ultrasonic power, and 1:2 (g:g) of EO/ethanol ratio. The results of SEM and FT-IR verified that the β-CD/EO IC was prepared successfully. TGA showed that the thermal stability of EO was increased obviously, and the dissolution experiment illustrated that the EO had a sustained-release function after encapsulation. Molecular docking and dynamic simulation revealed a dynamic equilibrium with a molar ratio of 1:1 between carvacrol and β-CD. Therefore, this study suggests that a two-step experimental design manifests a promising potential to screen out key parameters and the potential for developing new technologies combining experiments with molecular modeling.

## Figures and Tables

**Table 1:** Analysis of the significant parameters in the PBD test.

Item	Y_1_				Y_2_			
	Coefficient	Standard Error	T Value	*p* Value	Coefficient	Standard Error	T Value	*p* Value
Intercept	28.2173	1.1971	23.57	0.0001 **	6.6193	0.2481	26.68	0.0001 **
A	14.1787	2.8983	4.89	0.0018 **	−1.1420	0.3039	−3.76	0.0071 **
B	−0.2763	2.8983	−0.10	0.9267	0.1280	0.3039	0.42	0.6862
C	7.3563	2.8983	2.54	0.0388 *	0.3987	0.3039	1.31	0.2310
D	3.4867	2.6458	1.32	0.2290	0.5642	0.2774	2.03	0.0815
E	−2.9830	2.8983	−1.03	0.3376	−0.2553	0.3039	−0.84	0.4286
F	−6.2933	2.6458	−2.38	0.0490 *	−0.8375	0.2774	−3.02	0.0194 *
R^2^	0.8849 0.7698 9.1652				0.8113 0.6226 0.9600			
adjusted R^2^								
RMSE								

* *p* < 0.05, ** *p* < 0.01.

**Table 2:** Analysis of the significant parameters in the CCD test.

Item	Y_1_				Y_2_			
	Coefficient	Standard Error	T Value	*p* Value	Coefficient	Standard Error	T Value	*p* Value
Intercept	80.7578	1.5845	50.97	0.0001 **	9.0730	0.2145	42.29	0.0001 **
A	23.0624	0.9398	24.54	0.0001 **	−0.6435	0.1272	−5.06	0.0015 **
C	−0.1095	0.9398	−0.12	0.9106	−0.0623	0.1272	−0.49	0.6394
F	−1.5359	0.9398	−1.63	0.1462	−0.1098	0.1272	−0.86	0.4167
AC	1.8763	1.1176	1.68	0.1371	0.2538	0.1513	1.68	0.1374
AF	−2.6663	1.1176	−2.39	0.0485 *	−0.2913	0.1513	−1.92	0.0956
CF	1.2163	1.1176	1.09	0.3125	0.0763	0.1513	0.50	0.6298
A^2^	−14.0362	1.3025	−10.78	0.0001 **	−1.4668	0.1763	−8.32	0.0001 **
C^2^	−1.2371	1.3025	−0.95	0.3738	−0.0541	0.1763	−0.31	0.7679
F^2^	−1.1526	1.3025	−0.88	0.4056	−0.0782	0.1763	−0.44	0.6706
R^2^	0.9906 0.9785 3.1609				0.9373 0.8567 0.4280			
adjusted R^2^								
RMSE								

* *p* < 0.05, ** *p* < 0.01.

**Table 3:** Analysis of the significant parameters in the CCD test.

Number	Predicted Value		Actual Value		Relative Deviation (%)	
	Y_1_ (%)	Y_2_ (%)	Y_1_ (%)	Y_2_ (%)	Y_1_ (%)	Y_2_ (%)
1	85.52	9.03	85.33	8.78	−0.22	−2.77
2			86.87	9.03	1.58	0.01
3			86.29	8.95	0.91	−0.91
Average			86.17	8.92	0.01	−0.01

**Table 4:** Levels of the factors tested in PBD.

Factor	Symbol	Experimental Value		
		Low Level	Center Level	High Level
β-CD/EO ratio (g:g)	A	4 (−1)	8 (0)	12 (+1)
Ultrasonic time (min)	B	15 (−1)	30 (0)	45 (+1)
Ultrasonic power (W)	C	180 (−1)	270 (0)	360 (+1)
Temperature (°C)	D	40 (−1)	50 (0)	60 (+1)
Reaction water (mL:g)	E	8 (−1)	12 (0)	16 (+1)
EO/ethanol ratio (g:g)	F	0.5 (−1)	1.25 (0)	2 (+1)

**Table 5:** Design and results of PBD with Y_1_ and Y_2_ as responses.

Run	A/g:g	B/min	C/W	D/°C	E/mL:g	F/g:g	Y_1_ (%)	Y_2_ (%)
1	8	30	270	50	12	1.25	63.02	6.98
2	12	15	360	60	16	0.5	75.87	6.02
3	12	15	180	40	16	0.5	60.73	4.65
4	8	30	270	50	12	1.25	61.98	6.99
5	4	15	360	40	8	2	25.80	5.16
6	4	15	180	60	8	0.5	47.14	9.42
7	12	45	360	40	8	0.5	96.85	7.54
8	12	15	180	60	8	2	67.43	5.19
9	4	45	180	60	16	2	34.84	6.98
10	4	45	360	60	8	0.5	45.3	9.06
11	4	45	180	40	16	0.5	37.16	7.44
12	12	45	360	60	16	2	75.63	5.82
13	12	45	180	40	8	2	47.26	3.65
14	4	15	360	40	16	2	36.57	7.28
15	8	30	270	50	12	1.25	63.78	7.11

**Table 6:** CCD for optimization of the three significant factors.

Level	Experimental Value				
	−α	−1	0	+1	α
A (g:g)	2.85 (-α)	4 (−1)	8 (0)	12 (+1)	13.15 (α)
C (W)	154 (-α)	180 (−1)	270 (0)	360 (+1)	386 (α)
F (g:g)	0.28 (-α)	0.50 (−1)	1.25 (0)	2.00 (+1)	2.22 (α)

**Table 7:** Design and results of CCD with Y_1_ and Y_2_ as responses.

Run	A/g:g	C/W	F/g:g	Y_1_ (%)	Y_2_ (%)
1	8	270	0.28	82.41	9.31
2	12	360	2	86.52	6.66
3	4	180	0.5	41.67	8.31
4	4	360	0.5	38.92	7.77
5	8	270	1.25	79.96	8.96
6	12	360	0.5	90.09	7.04
7	8	270	1.25	77.59	8.72
8	8	154	1.25	82.38	9.25
9	12	180	2	76.90	5.88
10	8	386	1.25	78.24	8.85
11	12	180	0.5	90.16	6.90
12	4	180	2	43.90	8.79
13	8	270	2.22	78.49	8.71
14	4	360	2	41.19	8.22
15	13.15	270	1.25	91.32	6.45
16	8	270	1.25	80.42	9.08
17	2.85	270	1.25	26.90	6.97

## Data Availability

The data presented in this study are available on request from the corresponding author.

## References

[1] Li Z., Liu A., Du Q., Zhu W., Liu H., Naeem A., Guan Y., Chen L., Ming L. (2022). Bioactive substances and therapeutic potential of camellia oil: An overview. Food Biosci..

[2] Ming L., Huang H., Jiang Y., Cheng G., Zhang D., Li Z. (2019). Quickly identifying high-risk variables of ultrasonic extraction oil from multi-dimensional risk variable patterns and a comparative evaluation of different extraction methods on the quality of forsythia suspensa seed oil. Molecules.

[3] Zeroual A., Sakar E., Eloutassi N., Mahjoubi F., Chaouch M., Chaqroune A. (2020). Phytochemical profiling of essential oils isolated using hydrodistillation and microwave methods and characterization of some nutrients in *Origanum compactum* benth from central-northern morocco. Biointerface Res. Appl. Chem..

[4] Zeroual A., Sakar E., Eloutassi N., Mahjoubi F., Chaouch M., Chaqroune A. (2020). Wild chamomile [*Cladanthus mixtus* (L.) Chevall.] collected from central-northern morocco: Phytochemical profiling, antioxidant, and antimicrobial activities. Biointerface Res. Appl. Chem..

[5] Rao J., Chen B., McClements D.J. (2019). Improving the efficacy of essential oils as antimicrobials in foods: Mechanisms of action. Annu. Rev. Food Sci. Technol..

[6] Li Z., Jiang X., Huang H., Liu A., Liu H., Abid N., Ming L. (2022). Chitosan/zein films incorporated with essential oil nanoparticles and nanoemulsions: Similarities and differences. Int. J. Biol. Macromol..

[7] Li Z., Jiang X., Liu H., Yao Z., Liu A., Ming L. (2022). Evaluation of hydrophilic and hydrophobic silica particles on the release kinetics of essential oil Pickering emulsions. ACS Omega.

[8] Amalraj A., Haponiuk J.T., Thomas S., Gopi S. (2020). Preparation, characterization and antimicrobial activity of polyvinyl alcohol/gum arabic/chitosan composite films incorporated with black pepper essential oil and ginger essential oil. Int. J. Biol. Macromol..

[9] Yin H., Wang C., Yue J., Deng Y., Jiao S., Zhao Y., Zhou J., Cao T. (2021). Optimization and characterization of 1,8-cineole/hydroxypropyl-β-cyclodextrin inclusion complex and study of its release kinetics. Food Hydrocoll..

[10] Gharby S., Oubannin S., Ait Bouzid H., Bijla L., Ibourki M., Gagour J., Koubachi J., Sakar E.H., Majourhat K., Lee L.-H. (2022). An overview on the use of extracts from medicinal and aromatic plants to improve nutritional value and oxidative stability of vegetable oils. Foods.

[11] Tian Q., Zhou W., Cai Q., Ma G., Lian G. (2021). Concepts, processing, and recent developments in encapsulating essential oils. Chin. J. Chem. Eng..

[12] Assaba I.M., Rahali S., Belhocine Y., Allal H. (2021). Inclusion complexation of chloroquine with *α* and *β*-cyclodextrin: Theoretical insights from the new B97-3c composite method. J. Mol. Struct..

[13] Shankar V.K., Police A., Pandey P., Cuny Z.G., Repka M.A., Doerksen R.J., Murthy S.N. (2021). Optimization of sulfobutyl-ether-β-cyclodextrin levels in oral formulations to enhance progesterone bioavailability. Int. J. Pharm..

[14] Periasamy R. (2021). Cyclodextrin-based molecules as hosts in the formation of supramolecular complexes and their practical applications—A review. J. Carbohydr. Chem..

[15] Xiao Z., Zhang Y., Niu Y., Ke Q., Kou X. (2021). Cyclodextrins as carriers for volatile aroma compounds: A review. Carbohydr. Polym..

[16] Mura P. (2015). Analytical techniques for characterization of cyclodextrin complexes in the solid state: A review. J. Pharm. Biomed. Anal..

[17] Kumar R., Kaur K., Uppal S., Mehta S.K. (2017). Ultrasound processed nanoemulsion: A comparative approach between resveratrol and resveratrol cyclodextrin inclusion complex to study its binding interactions, antioxidant activity and UV light stability. Ultrason. Sonochem..

[18] Li X., Zhang Z.H., Qiao J., Qu W., Wang M.S., Gao X., Zhang C., Brennan C.S., Qi X. (2022). Improvement of betalains stability extracted from red dragon fruit peel by ultrasound-assisted microencapsulation with maltodextrin. Ultrason. Sonochem..

[19] Herrera A., Rodriguez F.J., Bruna J.E., Abarca R.L., Galotto M.J., Guarda A., Mascayano C., Sandoval-Yanez C., Padula M., Felipe F.R.S. (2019). Antifungal and physicochemical properties of inclusion complexes based on beta-cyclodextrin and essential oil derivatives. Food Res. Int..

[20] Boateng I.D., Yang X.-M. (2021). Process optimization of intermediate-wave infrared drying: Screening by Plackett–Burman; comparison of Box-Behnken and central composite design and evaluation: A case study. Ind. Crops Prod..

[21] Ma L., Wang L., Tang J., Yang Z. (2016). Optimization of arsenic extraction in rice samples by Plackett-Burman design and response surface methodology. Food Chem..

[22] Saad M., Tahir H. (2017). Synthesis of carbon loaded gamma-Fe_2_O_3_ nanocomposite and their applicability for the selective removal of binary mixture of dyes by ultrasonic adsorption based on response surface methodology. Ultrason. Sonochem..

[23] Wang P., Wang Z., Wu Z. (2012). Insights into the effect of preparation variables on morphology and performance of polyacrylonitrile membranes using Plackett–Burman design experiments. Chem. Eng. J..

[24] Nezhadali A., Mojarrab M. (2016). Computational design and multivariate optimization of an electrochemical metoprolol sensor based on molecular imprinting in combination with carbon nanotubes. Anal. Chim. Acta.

[25] Maqbool I., Akhtar M., Ahmad R., Sadaquat H., Noreen S., Batool A., Khan S.U. (2020). Novel multiparticulate pH triggered delayed release chronotherapeutic drug delivery of celecoxib-β-cyclodextrin inclusion complexes by using Box-Behnken design. Eur. J. Pharm. Sci..

[26] Dayana Priyadharshini S., Bakthavatsalam A.K. (2016). Optimization of phenol degradation by the microalga *Chlorella pyrenoidosa* using Plackett-Burman design and response surface methodology. Bioresour. Technol..

[27] Patil S.S., Jena H.M. (2015). Statistical optimization of phenol degradation by *Bacillus pumilus* OS1 using Plackett–Burman design and response surface methodology. Arab. J. Sci. Eng..

[28] Yenduri G., Costa A.P., Xu X., Burgess D.J. (2022). Impact of critical process parameters and critical material attributes on the critical quality attributes of liposomal formulations prepared using continuous processing. Int. J. Pharm..

[29] Fang M., Yu Z., Zhang W., Cao J., Liu W. (2022). Friction coefficient calibration of corn stalk particle mixtures using Plackett-Burman design and response surface methodology. Powder Technol..

[30] Iyyappan J., Bharathiraja B., Varjani S., PraveenKumar R., Muthu Kumar S. (2022). Anaerobic biobutanol production from black strap molasses using *Clostridium acetobutylicum* MTCC11274: Media engineering and kinetic analysis. Bioresour. Technol..

[31] Favre L.C., Rolandelli G., Mshicileli N., Vhangani L.N., Dos Santos Ferreira C., van Wyk J., Buera M.D.P. (2020). Antioxidant and anti-glycation potential of green pepper (*Piper nigrum*): Optimization of β-cyclodextrin-based extraction by response surface methodology. Food Chem..

[32] Li Y., Chen Y., Li H. (2017). Recovery and purification of cholesterol from cholesterol-β-cyclodextrin inclusion complex using ultrasound-assisted extraction. Ultrason. Sonochem..

[33] Liu P., Wang R., Kang X., Cui B., Yu B. (2018). Effects of ultrasonic treatment on amylose-lipid complex formation and properties of sweet potato starch-based films. Ultrason. Sonochem..

[34] Prakash Maran J., Mekala V., Manikandan S. (2013). Modeling and optimization of ultrasound-assisted extraction of polysaccharide from *Cucurbita moschata*. Carbohydr. Polym..

[35] Shrestha M., Ho T.M., Bhandari B.R. (2017). Encapsulation of tea tree oil by amorphous beta-cyclodextrin powder. Food Chem..

[36] Razavi S.M., Haghtalab A., Khanchi A.R. (2021). Optimization of vanadium(V) extraction by 2-ethyl-1-hexanol and the study of extraction reaction mechanism. Miner. Eng..

[37] Zhao L., Sun Q., Pu H., Tang P., Liu Y., Li M., Ren X., Li H. (2020). Experimental and computer simulation investigations of ethyl red with modified β-cyclodextrins: Inclusion mechanism and structure characterization. Chem. Phys. Lett..

[38] Shanmuga priya A., Balakrishnan S.b., Veerakanellore G.B., Stalin T. (2018). In-vitro dissolution rate and molecular docking studies of cabergoline drug with β-cyclodextrin. J. Mol. Struct..

[39] da Rocha Neto A.C., de Oliveira da Rocha A.B., Maraschin M., Di Piero R.M., Almenar E. (2018). Factors affecting the entrapment efficiency of β-cyclodextrins and their effects on the formation of inclusion complexes containing essential oils. Food Hydrocoll..

[40] Tahir M.N., Cao Y., Azzouz A., Roy R. (2021). Host-guest chemistry of the sulfasalazine-β-cyclodextrin inclusion complex. Tetrahedron.

[41] Garg A., Ahmad J., Hassan M.Z. (2021). Inclusion complex of thymol and hydroxypropyl-β-cyclodextrin (HP-β-CD) in polymeric hydrogel for topical application: Physicochemical characterization, molecular docking, and stability evaluation. J. Drug Deliv. Sci. Technol..

[42] Li Z., Jiang X., Yao Z., Chen F., Zhu L., Liu H., Ming L. (2022). Chitosan functionalized cellulose nanocrystals for stabilizing Pickering emulsion: Fabrication, characterization and stability evaluation. Colloids Surf. A.

[43] Gholibegloo E., Mortezazadeh T., Salehian F., Ramazani A., Amanlou M., Khoobi M. (2019). Improved curcumin loading, release, solubility and toxicity by tuning the molar ratio of cross-linker to β-cyclodextrin. Carbohydr. Polym..

[44] Liu R., Qin X., Liu X., Wang Y., Zhong J. (2022). Host-guest interactions between oleic acid and β-cyclodextrin: A combined experimental and theoretical study. Food Chem..

[45] Prabu S., Sivakumar K., Nayaki S.K., Rajamohan R. (2016). Host-guest interaction of cytidine in β-cyclodextrin microcavity: Characterization and docking study. J. Mol. Liq..

[46] Shabkhiz M.A., Khalil Pirouzifard M., Pirsa S., Mahdavinia G.R. (2021). Alginate hydrogel beads containing *Thymus daenensis* essential oils/glycyrrhizic acid loaded in β-cyclodextrin. Investigation of structural, antioxidant/antimicrobial properties and release assessment. J. Mol. Liq..

[47] Uppal S., Kaur K., Kumar R., Kahlon N.K., Singh R., Mehta S.K. (2017). Encompassment of benzyl isothiocyanate in cyclodextrin using ultrasonication methodology to enhance its stability for biological applications. Ultrason. Sonochem..

[48] Deng C., Cao C., Zhang Y., Hu J., Gong Y., Zheng M., Zhou Y. (2022). Formation and stabilization mechanism of β-cyclodextrin inclusion complex with C10 aroma molecules. Food Hydrocoll..

[49] Hu Z., Shao M., Zhang B., Fu X., Huang Q. (2022). Enhanced stability and controlled release of menthol using a β-cyclodextrin metal-organic framework. Food Chem..

[50] Sun Q., Tang P., Zhao L., Pu H., Zhai Y., Li H. (2018). Mechanism and structure studies of cinnamaldehyde/cyclodextrins inclusions by computer simulation and NMR technology. Carbohydr. Polym..

[51] Pradhan P.C., Mandal A., Dutta A., Sarkar R., Kundu A., Saha S. (2022). Delineating the behavior of *Berberis* anthocyanin/β-cyclodextrin inclusion complex in vitro: A molecular dynamics approach. LWT.

[52] Cid-Samamed A., Rakmai J., Mejuto J.C., Simal-Gandara J., Astray G. (2022). Cyclodextrins inclusion complex: Preparation methods, analytical techniques and food industry applications. Food Chem..

[53] Dos Santos Silva Araujo L., Lazzara G., Chiappisi L. (2021). Cyclodextrin/surfactant inclusion complexes: An integrated view of their thermodynamic and structural properties. Adv. Colloid. Interface Sci..

[54] Li Z., Ding Y., Huang H., Wang K., Wu J., Zhu L., Liao Z., Ming L. (2021). Study of β-cyclodextrin differential encapsulation of essential oil components by using mixture design and NIR: Encapsulation of α-pinene, myrcene, and 3-carene as an example. J. Chin. Pharm. Sci..

[55] Xiong W., Li Y., Ren C., Li J., Li B., Geng F. (2021). Thermodynamic parameters of gelatin-pectin complex coacervation. Food Hydrocoll..

[56] Jansook P., Ogawa N., Loftsson T. (2018). Cyclodextrins: Structure, physicochemical properties and pharmaceutical applications. Int. J. Pharm..

[57] Fateminasab F., Bordbar A.K., Shityakov S., Saboury A.A. (2020). Molecular insights into inclusion complex formation between β- and γ-cyclodextrins and rosmarinic acid. J. Mol. Liq..

[58] Rajendiran N., Siva S. (2014). Inclusion complex of sulfadimethoxine with cyclodextrins: Preparation and characterization. Carbohydr. Polym..

[59] Alshehri S., Imam S.S., Altamimi M.A., Jafar M., Hassan M.Z., Hussain A., Ahad A., Mahdi W. (2020). Host-guest complex of β-cyclodextrin and pluronic F127 with luteolin: Physicochemical characterization, anti-oxidant activity and molecular modeling studies. J. Drug Deliv. Sci. Technol..

[60] Chakraborty S., Karmakar A., Goswami T., Ghosh P., Mandal A. (2021). A combined spectroscopic and molecular dynamic analysis of the inclusion behaviour of L-serine and β-cyclodextrin. J. Mol. Liq..

[61] Al-Qubaisi M.S., Rasedee A., Flaifel M.H., Eid E.E.M., Hussein-Al-Ali S., Alhassan F.H., Salih A.M., Hussein M.Z., Zainal Z., Sani D. (2019). Characterization of thymoquinone/hydroxypropyl-β-cyclodextrin inclusion complex: Application to anti-allergy properties. Eur. J. Pharm. Sci..

[62] Ghorbel-Bellaaj O., Hmidet N., Jellouli K., Younes I., Maalej H., Hachicha R., Nasri M. (2011). Shrimp waste fermentation with *Pseudomonas aeruginosa* A2: Optimization of chitin extraction conditions through Plackett-Burman and response surface methodology approaches. Int. J. Biol. Macromol..

[63] Ming L., Li Z., Wu F., Du R., Feng Y. (2017). A two-step approach for fluidized bed granulation in pharmaceutical processing: Assessing different models for design and control. PLoS ONE.

[64] Li Z., Jiang X., Zhu L., Chen F., Liu H., Ming L. (2022). New insights into the thermal degradation behavior of hydroxypropyl-beta-cyclodextrin inclusion complexes containing carvacrol essential oil via thermogravimetric analysis. J. Therm. Anal. Calorim..

[65] Liu H., Yang G., Tang Y., Cao D., Qi T., Qi Y., Fan G. (2013). Physicochemical characterization and pharmacokinetics evaluation of β-caryophyllene/β-cyclodextrin inclusion complex. Int. J. Pharm..

[66] Zhu W.F., Zhu L., Li Z., Wu W.T., Guan Y.M., Chen L.H., Mao Z.X., Ming L.S. (2021). The novel use of PVP K30 as templating agent in production of porous lactose. Pharmaceutics.

[67] Shukla S.K., Chan A., Parvathaneni V., Kanabar D.D., Patel K., Ayehunie S., Muth A., Gupta V. (2020). Enhanced solubility, stability, permeation and anti-cancer efficacy of celastrol-β-cyclodextrin inclusion complex. J. Mol. Liq..

[68] Sursyakova V.V., Levdansky V.A., Rubaylo A.I. (2019). Thermodynamic parameters for the complexation of water-soluble betulin derivatives with (2-hydroxypropyl)-β-cyclodextrin determined by affinity capillary electrophoresis. J. Mol. Liq..

[69] Tambe A., Pandita N., Kharkar P., Sahu N. (2018). Encapsulation of boswellic acid with β- and hydroxypropyl-β-cyclodextrin: Synthesis, characterization, in vitro drug release and molecular modelling studies. J. Mol. Struct..

[70] Matencio A., Caldera F., Rubin Pedrazzo A., Khazaei Monfared Y., Dhakar N.K., Trotta F. (2021). A physicochemical, thermodynamical, structural and computational evaluation of kynurenic acid/cyclodextrin complexes. Food Chem..

[71] Menezes P.d.P., Serafini M.R., de Carvalho Y.M.B.G., Soares Santana D.V., Lima B.S., Quintans-Júnior L.J., Marreto R.N., de Aquino T.M., Sabino A.R., Scotti L. (2016). Kinetic and physical-chemical study of the inclusion complex of β-cyclodextrin containing carvacrol. J. Mol. Struct..

[72] Li Z., Wen W., Chen X., Zhu L., Cheng G., Liao Z., Huang H., Ming L. (2021). Release characteristics of an essential oil component encapsulated with cyclodextrin shell matrices. Curr. Drug Deliv..

[73] Makieła D., Janus-Zygmunt I., Górny K., Gburski Z. (2019). The dynamics of β-cyclodextrin molecules on graphene sheet. A molecular dynamics simulation study. J. Mol. Liq..

